# Effect of Housing Quality on the Mental Health of University Students during the COVID-19 Lockdown

**DOI:** 10.3390/ijerph19052918

**Published:** 2022-03-02

**Authors:** Alessandro Morganti, Andrea Brambilla, Andrea Aguglia, Andrea Amerio, Norberto Miletto, Nicolò Parodi, Chiara Porcelli, Anna Odone, Alessandra Costanza, Carlo Signorelli, Gianluca Serafini, Mario Amore, Stefano Capolongo

**Affiliations:** 1 Design & Health Lab, Department of Architecture, Built Environment and Construction Engineering (DABC), Politecnico di Milano, 20133 Milan, Italy or amorganti@stanford.edu (A.M.); andrea1.brambilla@polimi.it (A.B.); stefano.capolongo@polimi.it (S.C.); 2 Department of Psychiatry and Behavioral Sciences, Stanford University, Stanford, CA 94305, USA; 3 Department of Neuroscience, Rehabilitation, Ophthalmology, Genetics, Maternal and Child Health (DINOGMI), Section of Psychiatry, University of Genoa, 16132 Genoa, Italy; andrea.aguglia@unige.it (A.A.); andrea.amerio@unige.it (A.A.); norberto.miletto@gmail.com (N.M.); parodinicol@yahoo.com (N.P.); chiaraporcelli93@gmail.com (C.P.); mario.amore@unige.it (M.A.); 4 IRCCS Ospedale Policlinico San Martino, 16132 Genoa, Italy; 5 Department of Public Health, Experimental and Forensic Medicine, University of Pavia, 27100 Pavia, Italy; anna.odone@unipv.it; 6 Department of Psychiatry, Faculty of Medicine, University of Geneva (UNIGE), 1205 Geneva, Switzerland; alessandra.costanza@unige.ch; 7 School of Medicine, Vita-Salute San Raffaele University, 20132 Milan, Italy; signorelli.carlo@hsr.it

**Keywords:** COVID-19, lockdown, housing built environment, indoor quality, house dimension, mental health, evidence-based design

## Abstract

COVID-19 outbreak imposed rapid and severe public policies that consistently impacted the lifestyle habits and mental health of the general population. Despite vaccination, lockdown restrictions are still considered as potential measures to contrast COVID-19 variants spread in several countries. Recent studies have highlighted the impacts of lockdowns on the population’s mental health; however, the role of the indoor housing environment where people spent most of their time has rarely been considered. Data from 8177 undergraduate and graduate students were collected in a large, cross-sectional, web-based survey, submitted to a university in Northern Italy during the first lockdown period from 1 April to 1 May 2020. Logistic regression analysis showed significant associations between moderate and severe depression symptomatology (PHQ-9 scores ≥ 15), and houses with both poor indoor quality and small dimensions (OR = 4.132), either medium dimensions (OR = 3.249) or big dimensions (OR = 3.522). It was also found that, regardless of housing size, poor indoor quality is significantly associated with moderate–severe depressive symptomatology. Further studies are encouraged to explore the long-term impact of built environment parameter modifications on mental health, and therefore support housing and public health policies.

## 1. Introduction

### 1.1. COVID-19 Lockdown Measures and Impacts

The 2019 COVID-19 outbreak resulted in strict public policies that significantly affected lifestyle habits and the mental health of the global population [1,2,3]. In particular, before the important scientific advances in vaccine research, lockdown measures were adopted to restrict population movements and curb the SARS-CoV-2 virus spread. Such measures started in China in January 2020 and rapidly expanded to Europe, the Middle East, the USA, and the rest of the world, impacting lifestyle habits [4,5,6,7]. Italy, one of the first and hardest hit regions in Europe during the first COVID-19 wave, extended lockdown measures to the national territory starting from 11 March 2020 and released them only at the beginning of May 2020 [8,9,10].

Although the vaccination campaign in 2021 proceeded with significant achievements in many high-income countries [11,12,13], different governments and policymakers are still considering several lockdown measures as possible tools to limit the spread of COVID-19 variants [14]. The potential benefits of lockdown need to be carefully weighed against the possible impacts on people’s daily life and negative mental health effects, which are exacerbated by duration, difficulties caused by home confinement, fear of infection, frustration, boredom, inadequate supplies, financial loss, and stigma [15,16,17,18]. In fact, such measures emphasized already existing unequal vulnerabilities based on socio-demographic characteristics (e.g., job precarity, age, health, family composition) [19], but also housing inequalities (e.g., urban form, housing condition, affordability). The global pandemic required millions of people to considerably increase their time spent indoors, worsening mental health conditions, especially in individuals that experienced poor indoor quality settings [19]. COVID-19 significantly contributed to changed working habits too, with an increased adoption of working solutions that transformed home environments into work environments. In the scientific literature, several studies have highlighted the impact of COVID-19 and the lockdowns on the mental health and depression of students, one of the most fragile demographics of the population [20,21,22]. Nevertheless, the role of the housing environment where most of them spent their daily hours was rarely considered [23]. At the same time, the inadequate setting of several housing spaces for prolonged periods of mandatory working from home caused by lockdowns or quarantines has been explored [24].

### 1.2. The Role of the Housing Environment

The renewed attention to housing problems is linked to the fact that during lockdown periods, the daily time that the majority of citizens spent inside their home grew tremendously. Therefore, structural problems related to housing size and indoor quality of spaces emerged more clearly. Data from 2020 clearly showed that about 17.8% of the EU-27 population lived in inadequate and overcrowded dwellings [25], as defined by the number of rooms available and the household’s size, as well as its members’ ages and their family situation. Moreover, some reports showed trends towards smaller and less-flexible housing spaces [26]. With specific regard to the Italian population, recent data showed that the percentage of people living in overcrowded and outdated buildings was more concerning compared to the European average [27]. In fact, more than a third of the homes measured less than 60 m^2^, and among the bigger ones, overcrowding situations were frequent, leading to privacy reduction and an overall decrease in indoor environmental qualities [28].

Studies published in recent literature have started investigating, in a systematic way, the overall quality of built environment [29,30] and living spaces, revealing that the absence of accessible outdoor space from the house (e.g., garden, terrace) contributed to concerning levels of psychological and behavioral symptomatology [31], confirming that housing environments could be associated with the mental health and wellbeing of residents [32,33,34,35,36]. While mental illnesses can be investigated through structured and validated scales, housing quality is mainly assessed in terms of occupants’ perception, due to the complexity of surveying a high number of different apartments and the low psychometric reliability of existing tools [37,38,39,40,41].

### 1.3. Study Objective

Although a strong correlation of depression and anxiety symptoms with built environment has been reported by previous publication by the authors [31], built environment is often not considered relevant. A better insight on the issue is only possible by exploring every feature that composes the built environment. Therefore, investigation is needed to shed light on the role that indoor housing characteristics have on occupants’ well-being and mental health. The aim of this study was to deepen the knowledge on the relevance of indoor housing quality, according to depressive symptomatology during the first COVID-19 lockdown in Italy, through the support of data collected from a large sample of university students.

## 2. Materials and Methods

### 2.1. Sample

An online survey was sent by email from 1 April 2020 to 1 May 2020 to the personnel of a university in Milan, in the Lombardy region of Northern Italy. A total sample of N = 9261 full answers was gathered through the Google Forms platform, composing bachelor’s students, master’s students, PhD students, teaching staff, and administrative personnel, aged ≥18 years old. General data and descriptive statistics from the same survey were previously published in two scientific papers [31,42]. To ensure sample homogeneity and reduce biases, in the present study, only the student sub-population was selected for the analysis, including bachelor’s, master’s, and PhD students. No economic reward was provided to participants, and written consent was filled out before starting the survey. No identifiable information was collected, and participant’s anonymity and confidentiality were assured. Participants were able to resign from the study at any time.

### 2.2. Survey Questionnaire

The survey was structured in three sections, reflecting the areas of interest of the study. Question order and phrasing were carefully chosen so as not to prime participants for sensitive domains of investigation, or bias their answers. Participants were allowed not to answer some questions, or choose an “unknown” option, to avoid inaccurate data collection.

The first section collected general socio-demographic information of the participant, such as: (a) gender, (b) current age, (c) marital status, (d) educational level in years, (e) working position. The second section addressed questions based on international and validated clinical assessment scales, investigating depressive-, anxiety- and sleep-related symptoms, impulsivity, and quality of life. In the third section, we investigated housing physical characteristics through multiple choice and open questions.

In particular, this study focused on the relationship between depressive-related symptoms, housing size, and indoor housing characteristics.

For depressive-related symptomatology, the Patient Health Questionnaire (PHQ-9) scale was adopted [19], assessing the severity of depressive symptoms during the previous two weeks. Among a range score from 0 to 27, the severity could be assessed as normal (0–4), (2) mild (5–9), (3) moderate (10–14), (4) moderate/severe (15–19), and severe (20–27).

In the third section, the architectural parameters were clustered into:

- Housing typology (e.g., independent house, apartment); energy efficiency level (based on the energy efficiency score, defined by the Italian d.lgs. 19 August 2005, n. 192 and DPR 75/2013 regulations); structural renovation history from the last 10 years; dimension in terms of net square meters; number of rooms;
- View typology (nature or buildings) and subjective quality of views (poor or good);

Other variables were analyzed through the survey, such as: access to livable outdoor spaces (balcony or garden) measured in terms of balcony depth and garden access. If the respondent had access to outdoor spaces, natural daylight exposure of outdoor spaces was assessed in terms of hours per day; such variables are not pertinent for this paper, and were previously analyzed and discussed in a scientific publication by the authors [31,42].

Housing dimension was divided into three sub-categories, distinguishing small houses (<60 sqm) from medium houses (61–120 sqm) and big houses (>120 sqm).

An Indoor Quality Index (IQI) was defined by combining a set of parameters investigated by the survey: natural lighting, acoustic comfort, thermo-hygrometric comfort, need for artificial lighting during the day, presence/absence of soft qualities in the living area such as art objects or greenery/plants, and presence/absence of privacy during phone calls for work or personal reasons. Furthermore, combining the scores obtained, we considered the quality of indoor spaces as high (6 to 7 satisfied parameters), medium (4 to 5 satisfied parameters), or poor (0 to 3 satisfied parameters), as shown in Table 1.

### 2.3. Statistical Analysis

Statistical Package for Social Sciences (Version 25.0, SPSS; SPSS Inc., Chicago, IL, USA) for Windows was used for statistical analysis, and the significance was set at *p* < 0.05 (two-tailed).

Socio-demographic and clinical data, presented as means ± standard deviation (SD) or count and percentage, were evaluated for normal distribution by the Kolmogorov–Smirnov test. Bivariate and linear regression analyses were used to test the association between moderate–severe to severe depressive symptoms and poor indoor quality. Subsequently, Pearson’s chi-square test with Yates’ correction was employed for our analysis. For this analysis, we used a binary measurement for depression, based on PHQ-9 total score (absent or mild depression = 0; moderate or severe depression = 1). Finally, a logistic regression analysis was performed to explore the relationship between students reporting PHQ-9 ≥ 15 (dependent variable) and each of the other independent variables (architectural parameters) previously found to be associated in the statistical analysis, including gender and current age as covariates. The probability of entering the equation was set at 0.05.

## 3. Results

The survey was completed by 9261 participants, and most of the sample was represented by students (*n* = 8177, 88.3%). To have a homogeneous sample for the statistical analysis, only undergraduate and graduate students were considered (*n* = 8177) as reported in Table 2. The overall response rate (ORR) for the selected population was around 31.5%.

The selected sample, characterized by a male:female ratio, was 1:1.004 with a current mean age and an educational level of 22.02 ± 2.88 and 14.26 ± 1.68 years, respectively. The most relevant socio-demographic characteristics are reported in Table 3.

First, a significant association between moderate–severe to severe depressive symptoms and poor indoor quality was found in both the bivariate (r = 0.196, *p* < 0.001) and linear regression analysis (B = 0.181, SE = 0.010, t = 18.114, *p* < 0.001).

**Figure 1:**
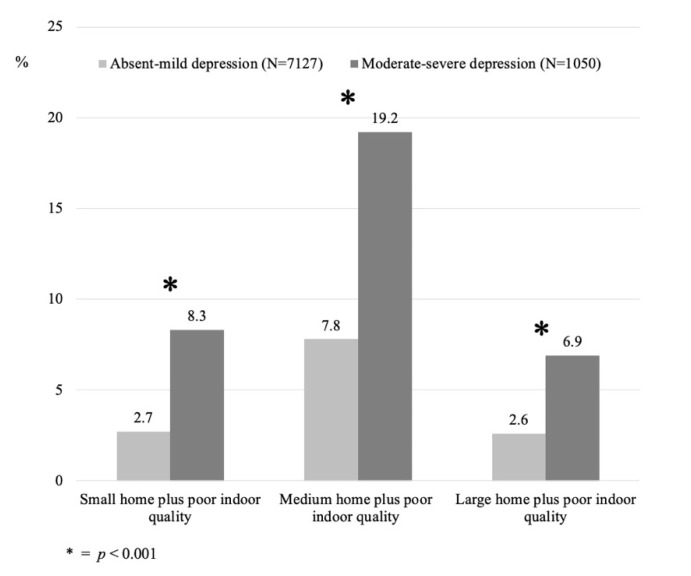
Prevalence of absent–mild and moderate–severe to severe depressive symptomatology among the three different subgroups studied.

Second, significant statistical associations were found between the population that reported moderate–severe to severe depressive symptoms and that had a poor indoor quality (8.3%), compared to the population with lower severity of depressive symptomatology (2.7%) for those living in small dwellings. Similar associations were found for medium (19.2%) and large (6.9%) apartments with poor IQI compared to the population with a lower severity of depressive symptomatology (7.8% and 2.6%, respectively) (see Figure 1).

**Figure 2:**
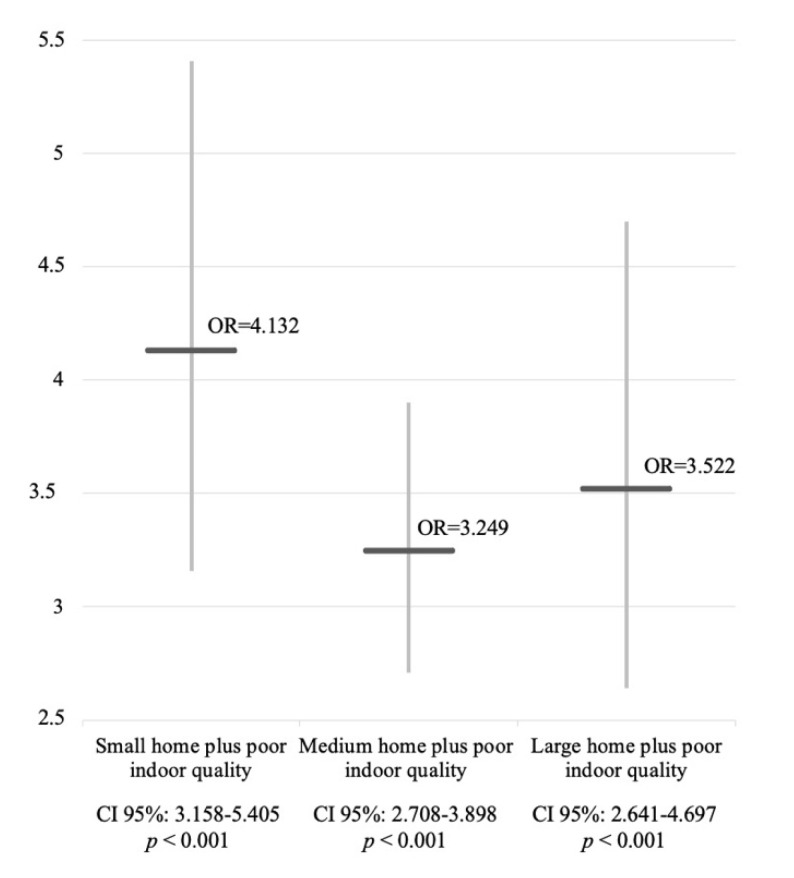
Comparison between ORs of the three subsamples of population.

From a logistic regression analysis, data showed significant statistical associations among the population with PHQ-9 scores ≥ 15 that were also living in homes with both poor indoor quality and small dimensions (odds ratio (OR) = 4.132, CI 95% = 3.158–5.405), as well as in houses of medium dimensions (OR = 3.249, CI 95% = 2.708–3.898) or big dimensions (OR = 3.522, CI 95% = 2.641–4.697) with poor indoor quality (low IQI), as shown in Figure 2.

## 4. Discussion

### 4.1. Poor Indoor Quality and Depressive Symptomatology in Student Population during COVID-19 Lockdown

Findings from the web-based, cross-sectional survey suggest significant associations between houses’ poor indoor quality and a higher severity of depressive symptoms, regardless of the housing dimensions. The appropriateness of the indoor housing characteristics, such as comfort, window view, and environmental qualities, were clearly identified as more impactful for inhabitant’s mental health rather than simply living in big, medium or small homes. A low IQI that grouped together low natural lighting, bad perceived acoustic comfort, scarce perceived thermo-hygrometric comfort, absence of soft qualities (e.g., art objects, green plants) and living spaces that did not guarantee adequate privacy (e.g., during phone calls for work or personal reasons), were mostly frequent in individuals with moderate–severe and severe depressive symptomatology compared to those with absent to moderate depressive symptomatology. These findings are consistent with the new challenges that COVID-19 lockdown measures brought to attention on housing environments, stressing the need for healthy, comfortable, and sustainable living places [46,48]. The data also emphasized the possible need for novel and more technological tools for flexible interior design modifications [49], common recreational open spaces, and vegetation views [20,50,51], as well as workspace adaptations [24,41]. Our findings also support a positive association between self-reported mental health symptomatology and indoor quality features, such as the presence of plant plots, or greater amounts of sunlight, as also reported in previous European studies [37,52,53].

Another relevant aspect highlighted by the study is the active participation of undergraduate and graduate students in such an investigation. A large audience was possible because of the online nature of the survey, and thus we were able to highlight the characteristics of this specific populational subgroup’s vulnerability and suffering during the first period of lockdown. University students experienced significant levels of stress, anxiety, and depression related to the pandemic; therefore, worsening their mental health conditions [54] and putting them at risk of long-term psychosocial consequences [55]. Specifically, students were required to continue their study activities while sharing homes 24 h a day with their families or roommates. Studies report that among the factors that most impacted student’s mental health was increased stress due to homework and lack of social interactions. As students were forced to perform multiple activities in the same space during different times of the day, specific features of the house—apart from size—may be important for them, as suggested by Farhan Asim and colleagues [47], such as the presence of indoor plants, and the view of natural elements from the window.

It is known that poor housing quality is associated with bad mental health development for children and adolescents [56], as well as family wellbeing [57]; findings from our study confirm that this is also true for older university students.

### 4.2. The Housing and Mental Health Dilemma: Is Quantity of Space Better than Quality?

Generally speaking, large apartment size could be imagined as the most important feature for students and other inhabitants, and therefore data about apartment dimension and density are used in the majority of national and international reports as indicators of critical housing conditions. Nevertheless, there is poor and contrasting evidence on how much housing size has an impact on mental health. As a wide systematic review on the topic by Charlotte Clark and colleagues reported [58], this is a challenging topic to explore: few longitudinal studies found an association between high housing density and inhabitant’s poor mental health. Other cross-sectional studies found no relevant associations at all, due to possible confounders such as socio-economic factors.

Although it is generally considered that bigger housing spaces could be an attractive feature for tenants, when it comes to the relationship of housing with increased depressive symptomatology and worse mental health conditions, our study highlights that the actual indoor quality of the apartment is more important to describe this phenomenon than size. For example, it is known that in living spaces, good lighting (both natural and artificial) is essential for safety and physical health, and greatly affects mental health too: studies showed that self-reported inadequate lighting increased the likelihood of depression [43]. Additionally, in confirmation of our findings, other studies found positive associations between indoor sound insulation and control with self-rated mental health in students [44,45].

All the above discussed indoor features are included in the IQI developed for the present study, and the results show that their presence in housing spaces can represent a synthetic and meaningful proxy of indoor quality that contributes to lower the prevalence of depressive symptomatology and poor mental health conditions. Starting from those results, a more vertical deepening of each specific built environment variable could provide important information for the development of safer and healthier housing environments. This will enable a better understanding of the direction of the relationship between built environment features and mental health, clarifying whether low-quality houses contribute to mental health or, conversely, depressed people may eventually be more likely to self-report inadequate features. The findings of this study open up relevant issues to be further deepened in future research.

## 5. Conclusions

Despite the perception of the importance of housing dimensions in coping with lockdown or quarantine measures, our findings reported that, regardless of housing size, poor indoor quality is the determinant that is most associated with more moderate/severe and severe depressive symptomatology in university students.

Social inequalities play a determining role on mental health, and contributing economic factors always have a direct impact on dwellers’ housing conditions, affecting indoor quality and size. Lockdown measures placed a magnifying lens on poor housing conditions, allowing our large sample study to confirm the crucial role of indoor housing quality on mental health indicators, as opposed to housing size.

As the pandemic amplified long-standing housing inequalities among the population, the presented findings should be seriously taken into account by national and local administrations to address more effective housing renovation policies, and resilience trajectories.

Indoor housing quality is notoriously difficult to investigate, but our novel findings come together with similar existing ones to propose some crucial indoor elements that have a proven impact on dwellers’ mental health, underlining the importance of further investigations on the topic.

### Limitations and Strengths for Future Developments

The study limitations include the fact that data collection was only performed in the first lockdown period, participants were recruited from just one university, and the absence of adjustment for socio-demographic confounders. Additionally, the main assumption of the study was that the direction of the relationship was from housing variables to mental health issues: further development will also need to explore the relevance of the opposite direction. The strengths of the study include a remarkably large population sample, and an early-stage reliable analysis on the consequences of the first COVID-19 lockdown that allowed us to study of the impact of living environments on mental health among university students.

Future studies are encouraged to further deepen the knowledge on crucial housing characteristics for mental health outcomes, forming a detailed comparison of specific variables with other studies, and to explore the long-term impacts of built environment parameters on the support for more effective housing and public health policies.

## Figures and Tables

**Table 1:** Examples of Indoor Quality Index (IQI) construction.

Indoor Quality Index (IQI)	COVID-19 Reference	High (Example)	Medium (Example)	Poor (Example)
Natural lighting	Osibona et al., 2021 [43]	X	X	
Acoustic comfort	Dzhambov et al., 2021 [44]; Torresin et al., 2022 [45]	X	X	X
Thermo-hygrometric comfort	D’Alessandro et al., 2020 [46]	X	X	
Artificial lighting during the day	Osibona et al., 2021 [43]	X		X
Art objects or greenery/plants	Asim et al., 2021 [47]	X	X	
Privacy during calls	Cuerdo-Vilches et al., 2021 [24];	X		
TOTAL		6 to 7 satisfied parameters	4 to 5 satisfied parameters	0 to 3 satisfied parameters

**Table 2:** Population sample divided by working roles.

Working Role	*n* (%)
Professors	266 (2.9)
PhD student	443 (4.7)
Non-doctorate student	8177 (88.3)
Administrative staff	376 (4.1)

**Table 3:** Socio-demographic characteristics of the total sample included.

Characteristics	Total Sample (*n* = 8177)
Gender (females), *n* (%)	4082 (49.9)
Current age, mean ± SD	22.02 ± 2.88
Marital Status, *n* (%)	
Single	7999 (97.8)
Married	174 (2.1)
Separated/divorced	4 (0.1)
Educational level, mean ± SD	14.26 ± 1.68
